# From Pornography to Somatic Symptoms. A Moderated Mediation Model Involving Moral Disapproval of Pornography and Difficulties in Emotion Regulation

**DOI:** 10.1080/19317611.2026.2646620

**Published:** 2026-03-27

**Authors:** Tudor-Daniel Huțul, Adina Karner-Huțuleac, Antonia-Ștefania Petroiu, Florentina-Brigita Chiricheș, Andreea Huțul, Maria Drăgan, Ana Maria Ciobanu

**Affiliations:** a Department of Psychology, Faculty of Psychology and Education Sciences, Alexandru Ioan Cuza University of Iași, Iași, Romania; b Department of Education Sciences, Faculty of Psychology and Education Sciences, Alexandru Ioan Cuza University of Iași, Iași, Romania; c Department of Communication and Social Research, Sapienza University of Rome, Rome, Italy

**Keywords:** Problematic pornography consumption, somatic symptoms, difficulties in emotion regulation, moral disapproval of pornography, moderated mediation

## Abstract

**Objective:**

The present study aimed to examine the association between problematic pornography consumption (PPC) and somatic symptoms, and to investigate whether difficulties in emotion regulation and moral disapproval of pornography play a role in this relationship.

**Method:**

A sample of 321 individuals, aged between 18 and 64 years (M = 24.85, SD = 8.93), completed self-report measures assessing problematic pornography consumption, difficulties in emotion regulation, somatic symptoms, frequency of pornography use, and moral disapproval of pornography.

**Results:**

PPC was positively associated with somatic symptoms and difficulties in emotion regulation. Difficulties in emotion regulation mediated the association between PPC and somatic symptoms only at high levels of moral disapproval, indicating a moderated mediation effect. No significant indirect effect was observed at low or average levels of moral disapproval.

**Conclusions:**

These findings highlight the importance of integrating moral, emotional, and somatic dimensions when conceptualizing problematic pornography consumption. The role of moral disapproval suggests that individuals experiencing moral conflict may be particularly vulnerable to emotion regulation difficulties and somatic manifestations linked to pornography use, offering useful implications for future theory and clinical practice.

## Introduction

According to the fifth edition of the *Diagnostic and Statistical Manual of Mental Disorders* (American Psychiatric Association, 2013), somatic symptoms are expressions of individual distress shaped by cultural and social contexts. Somatization is defined in the literature as a secondary reaction to psychological distress (presenting somatization) or as a primary phenomenon characterized by symptoms without a clear medical explanation (functional somatization) (De Gucht & Fischler, 2002). More specifically, secondary somatization refers to physical symptoms that are a response to an existing mental health disorder, such as depression or anxiety, or a reaction to traumatic events (De Gucht & Fischler, 2002). In comparison, functional somatization refers to symptoms that cannot be explained by a medical disease or a clear cause, but are instead connected to a dysfunction of body functioning, which significantly affects daily life (De Gucht & Fischler, 2002).

In terms of the classification, Zeng et al. (2016) introduced and validated a theoretical model from the perspective of psychosomatic medicine, which divides these symptoms into four dimensions: emotional, biological, imagination-related, and cognitive somatic symptoms. For example, in difficult moments, individuals may respond with physiological reactions that are manifest as somatic symptoms, such as fatigue, headache, body pain, changes in appetite or gastrointestinal issues (Zijlema et al., 2013). Moreover, somatic symptoms frequently appear in chronic noninfectious conditions, where there is an interaction between the biological processes, emotional distress, and psychosocial mechanisms (Zeng et al., 2016). For a better understanding of the classification, it must be taken into consideration that symptoms not only reflect psychological suffering but also have negative effects such as difficulty in daily functioning, including work performance and family relationships, ultimately reducing overall quality of life (Liao et al., 2019).

Previous research has identified multiple predisposing factors for somatic symptoms such as comorbid depression and anxiety, and exposure to stressful or traumatic life events (De Gucht & Maes, 2006; Henningsen et al., 2003; Zheng et al., 2019). In addition to these predisposing factors, recent research indicates that there may be a potential link between problematic pornography consumption, typically conceptualized as a persistent and dysregulated pattern of pornography use associated with clinically significant distress and/or functional impairment (Bőthe et al., 2019; Grubbs & Perry, 2019; Huțul, et al., 2025a; Kraus et al., 2015), and somatic symptoms, particularly in the absence of adequate coping mechanisms and the presence of emotional dysregulation (Testa et al., 2024; Wang et al., 2024). Additionally, Chen et al. (2018) stated that problematic pornography consumption is connected to throbbing headaches especially at vertex, neck and bilateral temple. The same study also shows a link between problematic pornography consumption and other somatic symptoms like palpitation, rapid breathing, facial hotness and dry throat, stating that these appear before the headaches (Chen et al., 2018). Studies have also shown a wide range of other somatic symptoms, linked to withdrawal from pornography consumption, like: irritability, apathy, sleeping difficulties, headaches, sweating, chills, sickness, fatigue, restlessness, attention problems, libido reduction (reported during periods of abstinence or reduced sexual interest following cessation of pornography use), aggressiveness, hand trembling, drowsiness, strong heartbeats, muscle problems, pain in other body parts, stomachaches and nausea (Blinka et al., 2022; Cuppone et al., 2021; Darshan et al., 2014; Dwulit & Rzymski, 2019; Fernandez et al., 2021; Roza et al., 2024; Skryabin et al., 2021).

To conclude, given the ample negative outcomes associated with somatic symptoms, it becomes essential to further investigate factors that may contribute to their occurrence. Although recent findings suggest a potential link between problematic pornography consumption and somatic symptomatology—particularly in the context of inadequate coping mechanisms and emotional dysregulation (Testa et al., 2024; Wang et al., 2024)—the evidence remains scarce and fragmented. Existing studies report isolated associations, such as headaches, palpitations, rapid breathing, or other physiological reactions connected either to problematic use or withdrawal (Blinka et al., 2022; Cuppone et al., 2021; Darshan et al., 2014; Dwulit & Rzymski, 2019; Fernandez et al., 2021; Roza et al., 2024; Skryabin et al., 2021). However, these observations have not yet been systematically examined, leaving the direct relationship between problematic pornography consumption and somatic symptoms largely unexplored. In this context, the present study aims to assess the stability and strength of this potential association, addressing an important gap in the existing literature.

## Problematic pornography consumption and somatic symptoms

The widespread accessibility of the internet has provided individuals with unprecedented opportunities to engage with a wide range of information, communication and entertainment (Adorjan et al., 2021). In Romania, data published in early March 2025 indicates that 17.8 million individuals were using the internet, representing an online penetration of 94% (Kemp Simon, 2025). Together with the increase in internet accessibility, the online pornography industry has grown as well, both in terms of content availability and global consumption patterns (Lewczuk et al., 2022; Mengzhen et al., 2025; Owens et al., 2012). Pornography refers to sexually explicit films, video clips, or pictures displaying the genital area that are intended to sexually arouse the viewer and may be accessed through various media, including the internet, magazines, books, or television (Grubbs et al., 2022). Among the most common online activities (Brand et al., 2016, 2019, 2025), pornography consumption has been reported as highly prevalent across different populations. For example, among young Australians, exposure is widespread, with 86% of males and 69% of females having viewed pornography, and 54% of young men comparative 14% of young women reporting weekly use (Crabbe et al., 2024). The World Health Organization (2022) includes problematic Pornography Consumption within the *International Classification of Diseases*, 11th Revision (ICD-11), as part of Compulsive Sexual Behavior Disorder (CSBD), which is classified under impulse control disorders. When it comes to CSBD symptoms whom may also be expressed as “a variety of behaviors, including sexual behavior with others, masturbation, use of pornography, cybersex (internet sex), telephone sex, and other forms of repetitive sexual behavior”. In addition to this, a lot of individuals who experience CSBD symptoms reported a history of risky sexual behavior, excessive use of pornography and masturbation as a coping mechanism for negative emotions (*World Health Organization*, 2022).

Pornography consumption may carry both positive and negative consequences. While for some individuals pornography consumption may serve a recreational or exploratory purpose, such as enhancing sexual curiosity or contributing to sexual education (Hald & Malamuth, 2008; Litsou et al., 2021; Rothman et al., 2015), for others it can have negative consequences including relationship dissatisfaction, compulsive patterns of use, and psychological distress (Egan & Parmar, 2013; Grubbs, Volk, et al., 2015). This difference occurs because some individuals can reach problematic pornography consumption (Kraus et al., 2018). Furthermore, an important aspect is that the frequency of pornography consumption does not automatically indicate a problematic use, because some individuals can view pornography regularly and don’t feel a negative impact, while some individuals can consume less and report distress (Bőthe et al., 2020; Wright et al., 2022).

Despite significant progress in the area of study of problematic pornography consumption, the emergence of somatic symptoms remains a critical issue due to its implications for mental health, which is important to take into consideration because it affects daily life functioning (Nadinda et al., 2024). Although several mechanisms—particularly emotional dysregulation—have been associated with problematic pornography consumption (Mattebo et al., 2018), the extent to which these mechanisms may contribute to somatic manifestations is still unclear.

The literature on somatization suggests that aspects such as fatigue, physiological tension, sleep disturbances, or cardiopulmonary discomfort tend to occur together with high levels of stress or internal distress (Chen et al., 2018; Testa et al., 2024; Zeng et al., 2016; Zheng et al., 2019). Considering that problematic pornography consumption is often accompanied by shame, anxiety, and psychological distress (Grubbs, Stauner, et al., 2015; Huțul & Karner-Huțuleac, 2023), it is reasonable to assume that these emotional correlates could also increase vulnerability to somatic symptomatology.

However, even with these indications, the direct relationship between problematic pornography consumption and somatic manifestations remains insufficiently studied. Existing suggestions in the literature point toward a potential link, yet empirical evidence is scarce, fragmented, and lacks systematic investigation. This gap is particularly important, as understanding whether—and how—problematic pornography consumption contributes to somatic symptoms would offer a more comprehensive picture of its psychological and physiological impact. Therefore, the limited exploration of this association represents a significant gap in the current literature and highlights the need for further empirical research.

## Problematic pornography consumption and difficulties in emotion regulation

One of the concepts tightly linked to problematic pornography consumption is difficulties in emotional regulation (DERS; Cardoso et al., 2022, 2023). This has been conceptualized as involving the awareness, understanding and acceptance of emotions but also the ability to control impulsive behaviors and behave in accordance with desired goals when experiencing negative emotions (Gratz & Roemer, 2004). Difficulties in emotion regulation have been explored and defined in the literature as the inability of an individual to recognize, understand and effectively manage their emotions in challenging life situations or while maintaining focus on goal-directed behavior, particularly during negative emotional experiences (Bjureberg et al., 2016; Dan-Glauser & Scherer, 2013; Hallion et al., 2018).

When it comes to connection between emotion regulation and pornography including problematic pornography consumption Cardoso et al. (2022a) talked about how people experiencing higher emotional dysregulation will also have an increase in their pornography consumption. This information is corroborated by Levin et al. (2019) who conducted a study about the reasoning behind why people use pornography and found that the highest predictor for pornography viewing was emotional avoidance but at the same time emotional regulation and efficient coping strategies were strongly correlated to the severity of problematic pornography consumption (Antons et al., 2023).

Moreover, although much of the existing literature focuses on how difficulties in emotion regulation may predict or intensify problematic pornography consumption (Cardoso et al., 2022, 2023), several theoretical perspectives suggest that this relationship may also operate in the opposite direction. Repeated engagement in problematic pornography consumption—especially when employed as a maladaptive coping strategy (Bőthe et al., 2019; Grubbs & Perry, 2019; Kraus et al., 2015)—may, over time, undermine emotional processing capacities, reinforcing avoidance-based mechanisms and limiting the individual’s ability to tolerate distress or regulate internal states effectively (Antons et al., 2023; Brand et al., 2025; Starcke et al., 2018). In this sense, problematic pornography consumption could contribute to a worsening of emotion regulation difficulties, suggesting a potentially bidirectional relationship that has received limited empirical attention and warrants further investigation.

## The potential indirect effect of difficulties in emotion regulation

Difficulties in emotion regulation may act as a predictor both for addictive potential (Shirazi & Janfaza, 2015) and for pornography consumption (Cardoso et al., 2022). Shirazi and Janfaza (2015) found that addictive potential was positively associated with several dimensions of emotion dysregulation—such as difficulties engaging in goal-directed behavior, limited access to emotion regulation strategies, impulse control difficulties, and lack of emotional awareness and clarity—and negatively associated with self-control. These results suggest that individuals with low self-control are more likely to pursue immediate gratification (e.g., alcohol or drug use) as a strategy to regulate emotional distress (Shirazi & Janfaza, 2015).

According to Wizła and Lewczuk (2024), difficulties in emotion regulation mediated the relationship between attachment insecurity and both compulsive sexual behavior disorder and problematic pornography consumption. This finding is aligned with extensive literature showing that pornography may function as a maladaptive coping mechanism in situations where individuals struggle to regulate their emotions; through such use, pornography consumption may progressively become problematic and develop into an impulsive and persistent pattern of behavior (Testa et al., 2024)

At the same time, problematic consumption of pornography has been linked to a range of somatic symptoms, such as headaches, palpitations, and facial hotness (Chen et al., 2018). Taken together, these arguments support the hypothesis that difficulties in emotion regulation may serve as a mediator in the relationship between problematic pornography consumption and somatic symptoms. This study extends previous findings by examining how emotion regulation difficulties may explain the pathway from problematic pornography consumption to somatic responses. For example, an individual who engages in problematic pornography consumption may experience increasing difficulties in regulating emotional states, which, in turn, can heighten stress reactivity and contribute to the emergence of somatic symptoms.

## The moderating role of moral disapproval of pornography

Pornography consumption is often associated with internal conflicts, particularly when it comes to personal values and moral standards that are perceived to be in contradiction with the behavior itself (Grubbs & Perry, 2019; Huțul et al., 2025a; Huțul & Karner-Huțuleac, 2024b). Such discrepancies may lead to significant psychological distress, which can further contribute to using pornography in a dysfunctional and problematic way (Grubbs, Volk, et al., 2015; Huțul et al., 2024, 2025a; Testa et al., 2024). This idea is best explained by the Pornography Problems due to Moral Incongruence (PPMI; Grubbs et al., 2019), developed to better understand why individuals may perceive their pornography consumption as harmful, problematic, or even addictive, and which factors contribute to this perception. The PPMI model proposes two main pathways through which pornography-related problems may emerge: one pathway in which pornography problems directly lead to distress through dysregulation, and a second pathway in which moral incongruence plays a central role, with moral disapproval of pornography contributing directly to the experience of distress (Grubbs et al., 2019).

As indicated by the PPMI model, a particularly important factor influencing pornography-related distress is morality (Grubbs, Exline, et al., 2015). Consuming pornographic content despite morally disapproving of one’s own pornography consumption can lead to moral incongruence (Grubbs et al., 2019; Huțul & Karner-Huțuleac, 2024b). Moral incongruence occurs when one’s moral standards are perceived to be in contradiction with the behavior itself and is described as the interaction between the frequency of the behavior and the moral disapproval of that behavior (Grubbs et al., 2022; Grubbs & Perry, 2019; Huțul et al., 2025a). Importantly, moral disapproval of pornography is not conceptualized in the present study as a peripheral contextual factor, but rather as a central psychological mechanism through which pornography-related distress may be amplified and translated into emotional and somatic manifestations.

Problematic pornography consumption is considered part of compulsive sexual behavior disorder (CSBD), and prior research suggests that when people attach strong moral judgments to their sexual behavior, or are concerned about the moral judgment of others, they are more likely to perceive their pornography consumption as harmful, addictive, or compulsive (*World Health Organization*, 2022). Over the course of recent research, problematic pornography consumption has been repeatedly described as a maladaptive coping mechanism in situations involving intense emotions and psychological distress (Cardoso et al., 2023; Musetti et al., 2022; Testa et al., 2024; *World Health Organization*, 2022). At the same time, the literature suggests that maladaptive emotion regulation strategies are linked to poorer physical health and to the occurrence of somatic symptoms (Jungmann et al., 2022). Thus, moral disapproval of pornography may play a significant role in this relationship through the mechanism of moral incongruence, which can generate higher levels of distress, shame, and negative emotions that may, in turn, contribute to the emergence of somatic symptoms (Grubbs et al., 2019; Grubbs & Perry, 2019; Jungmann et al., 2022).

Furthermore, previous findings indicate that moral disapproval can enhance the relationship between problematic pornography consumption and psychological distress (Lewczuk et al., 2020). Concurrently, other studies have shown that moral disapproval mediates and amplifies the association between problematic pornography consumption and religiosity, with the strength of the relationship increasing as moral disapproval grows (Grubbs et al., 2019). This is particularly relevant given that Romania has a strong religious background, which maintains a taboo perspective on pornography consumption and sexuality and sustains a high level of moral judgment regarding this topic (Gergely & Rusu, 2023; Huțul & Karner-Huțuleac, 2023; Turcescu & Stan, 2005).

Considering the body of research findings and the Romanian cultural context, we expect that moral disapproval of pornography will moderate the association between problematic pornography consumption and somatic symptoms via difficulties in emotion regulation. Specifically, we anticipate that when moral disapproval is high, the overall relationship between problematic pornography consumption, emotion regulation difficulties, and somatic symptoms will be stronger, whereas at low levels of moral disapproval this association will be weaker.

## The present study

The aim of the present study is to advance the understanding of problematic pornography consumption by assessing its direct relationship with somatic symptoms. To our knowledge, no prior research has examined the general somatic implications of problematic pornography consumption, making this an important and largely unexplored area in the literature.

**Figure 1.**
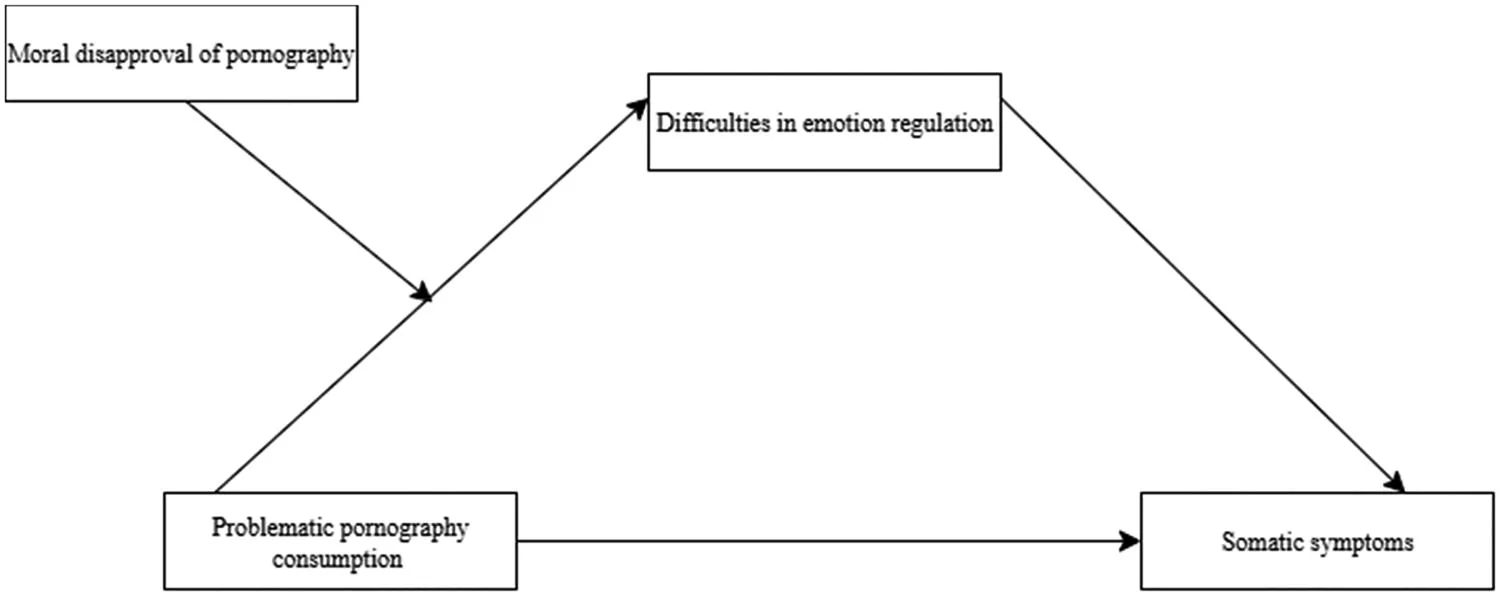
The proposed model.

Our main objective was to investigate whether problematic pornography consumption predicts somatic symptoms. We propose that individuals may resort to pornography consumption as a maladaptive coping mechanism when experiencing psychological distress and difficulties in emotion regulation, which in turn may increase the likelihood of somatic symptomatology. Moreover, we expect that this relationship will be stronger among individuals who morally disapprove of pornography consumption, given the emotional and cognitive conflict described in the literature on moral incongruence. Exploring these relationships is particularly relevant, as the findings may offer valuable insight for clinical psychologists, psychotherapists, and other mental health professionals working with individuals presenting problematic pornography consumption. Additionally, because frequency of pornography consumption is known to correlate with PPC, we included it as a control variable. To examine the proposed moderated mediation model—where problematic pornography consumption predicts somatic symptoms indirectly through difficulties in emotion regulation (DERS), and moral disapproval moderates the path from PPC to DERS (see Figure 1)—we formulated the following hypotheses:

## Hypotheses

H1. There are significant associations between problematic pornography consumption, difficulties in emotion regulation, somatic symptoms, frequency of pornography consumption, and moral disapproval of pornography.

H2. There is an indirect effect of difficulties in emotion regulation on the relationship between problematic pornography consumption and somatic symptoms, while controlling for the frequency of pornography consumption.

H3: Moral disapproval of pornography moderates the relationship between problematic pornography consumption and somatic symptoms via difficulties in emotion regulation, while controlling for the frequency of pornography consumption. We expect that this relationship will be stronger and statistically significant at higher levels of moral disapproval.

## Method

### Participants and procedure

The sample consisted of 321 participants who were not compensated for their involvement, making participation entirely voluntary. The mean age was 24.85 years (SD = 8.93), ranging from 18 to 64. Sociodemographic characteristics of the sample are presented in Table 1.

**Table 1.** The characteristics of the participants.

		N	%
Sex	Female	260	81.0
	Male	56	17.4
	Non-binary	5	1.6
Residence	Urban area	204	63.6
	Rural area	117	36.4
Studies	Pre-universitary	43	13.4
	University in progress	170	53.0
	Post-universitary	108	33.6
Current marital status	Single (not widowed or divorced)	133	41.4
	Married	34	10.6
	In a relationship (not married)	140	43.6
	Widowed (not in a relationship)	2	.6
	Divorced (not in a relationship)	6	1.9
	Prefer not to say	6	1.9
Personal income	Below €200 (approx. <1,000 RON/month)	74	23.1
	€200 – €499 (approx. 1,000–2,500 RON/month)	83	25.9
	€500 – €999 (approx. 2,500–5,000 RON/month)	64	19.9
	€1,000 – €1,999 (approx. 5,000–10,000 RON/month)	38	11.8
	€2,000 – €4,999 (approx. 10,000–25,000 RON/month)	8	2.5
	€5,000 – €9,999 (approx. 25,000–25,000 RON/month)	0	0
	€10,000 or more (approx. >50,000 RON/month)	3	.9
	I don’t want to answer	51	15.9
Religion	Orthodox	195	60.7
	Catholic	53	16.5
	Atheist	26	8.1
	Agnostic	29	9.0
	Other	18	5.5
Total		321	100

The study was conducted using an online survey administered via Google Forms. The survey link was distributed through several social media platforms, including Facebook, Instagram, Reddit, and Discord. To obtain participants’ informed consent, the first page of the survey presented detailed information about the study’s purpose, confidentiality, data protection, voluntary participation, and the right to withdraw at any time. Participants were required to read this information and indicate their agreement by checking a consent box. Contact information for the project coordinator was also provided for any questions regarding the study.

Recruitment targeted a broad community sample rather than a specific group, with the survey shared across various general-interest online communities (e.g., residents of Iași, Ploiești, Vaslui). This approach was intended to reduce the likelihood of obtaining responses from a single category of participants. Participation was open to all adults regardless of their educational status; therefore, the sample included individuals with pre-university education, those currently enrolled in university studies, as well as individuals who had completed post-university education. Data collection took place between February 11th and March 26th, 2025.

The only inclusion criterion was being at least 18 years old at the time of participation. No additional exclusion criteria were applied, and participants were not excluded based on sex, educational status, or pornography use patterns. The average time required to complete the survey was approximately 15–20 minutes.

All participants provided informed consent prior to beginning the survey. The study received full ethical approval from the Ethics Committee of the Faculty to which the authors are affiliated (No. 390/10.02.2025).

### Measures

To ensure precise translation of all instruments used, we utilized the back-translation method and followed recommendations from relevant literature on instrument translation and adaptation (Hambleton & Zenisky, 2010; Maneesriwongul & Dixon, 2004; Sousa & Rojjanasrirat, 2011).

Two teams of psychologists, experts in the studied variables, were involved. Each team independently translated the instruments, and the two resulting versions were compared and discussed to reach a final consensual version. Subsequently, a professional translator with expertise in psychology, who was not involved in the initial translation process and had no access to the original source, performed the back-translation of the final Romanian version into English. Any discrepancies identified between the back-translated version and the original were discussed and resolved to obtain the final version of the instruments. Through this method, we ensured the quality and fidelity of the translation, as well as the preservation of the intellectual meaning of the original measures.

Given the variability in how pornography is defined across studies, conceptual consistency was ensured by providing participants with a standardized definition prior to completing the relevant measures. Specifically, participants were presented with a definition adapted from Grubbs et al. (2022), in line with methodological recommendations emphasizing the importance of clear operationalization in pornography research (Kohut et al., 2020): “Pornography refers to any sexually explicit films, video clips or pictures displaying the genital area, which is intended to sexually arouse the viewer; this may be seen on the internet, in a magazine, in a book, or on television.” This definition has been employed in previous research examining pornography consumption and related psychological processes, supporting its conceptual clarity and applicability (Huțul et al., 2024, 2025a; Petroiu et al., 2025).

#### Somatic symptoms

To assess this construct, we used „The Somatic Stress Response Scale” (SSRS; Koh et al., 2005). This instrument is a self-report scale used for assessing stress related somatic symptoms, it contains 32 items comprising 5 factor categories: cardiorespiratory responses which contains eleven items (e.g., “I have chest pains”), somatic sensitivity containing five items (e.g., “I get rashes or skin blotches”), gastrointestinal responses containing eight items (e.g., “I have heartburn”), general somatic responses containing four items (e.g., “I feel my blood pressure rising”) and genitourinary responses also containing four items (e.g., “I have a decreased sex drive”). The responses are rated on a five-point Likert scale spanning from 0 “Not at all” to 4 “Absolutely”. The total score is obtained by summing all item scores, ranging from 32 to 160. Higher scores indicate a higher level of somatic symptoms. The internal consistency for SSRS in the present study was excellent (Cronbach’s α = 0.94).

#### Problematic pornography consumption

To assess this construct, we used “The Problematic Pornography Consumption Scale” (PPCS; Bőthe et al., 2018). This instrument contains 18 items organized into 6 factor categories, measuring salience (e.g., “I thought about how good it will be to watch porn”), mood modification (e.g., “Watching porn got rid of all my negative feelings”), conflict (e.g., “I feel porn causes problems in my personal life”), tolerance (e.g., “I gradually watched more “extreme” porn, because the porn I watched before was less satisfying”), relapse (e.g., “I unsuccessfully tried to reduce the amount of porn I watch”), and withdrawal (e.g., “I became agitated when I was unable to watch porn”). The responses are rated on a seven-point Likert scale spanning from 1 “Never” to 7 “Always”. The total score for this instrument is calculated by summing the scores obtained at all the 18 items. Higher scores mean a higher and more problematic consumption of pornography. This instrument has also demonstrated its psychometric properties on the Romanian population (Huțul et al., 2024; 2025a). The internal consistency for PPCS in the present study was excellent (Cronbach’s α = 0.94).

#### Difficulties in emotion regulation

To assess this construct, we used the “Difficulty in Emotional Regulation Scale” (DERS; Gratz & Roemer, 2004). This instrument contains 36 items organized into six subscales, measuring nonacceptance of emotional responses (e.g. “When I’m upset I feel guilty for feeling that way”), difficulty in engaging in goal-directed behaviors (e.g., “When I’m upset, I have difficulty getting work done”), impulse control difficulties (e.g., “When I’m upset, I lose control over my behaviors”), lack of emotional awareness (e.g., “I pay attention to how I feel.”), limited access to emotion regulation strategies (e.g., “When I’m upset, I believe that wallowing in it is all I can do”) and lack of emotional clarity (e.g., “I am confused about how I feel”). The responses are rated on a five-point Likert scale, from 1 – “almost never” to 5 – “almost always”. The total score is obtained by summing all item scores (36–180), with higher scores indicating greater difficulties in emotion regulation. In Romania this instrument has shown significant psychometric properties (Antoniac et al., 2025; Matei-Mitacu et al., 2024). The internal consistency for DERS in the present study was excellent (Cronbach’s α = 0.89).

#### Moral disapproval of pornography

To measure this construct, we used four items (e.g., “I believe that viewing pornography online weighs on my conscience”). These items were rated on a seven-point Likert scale, spanning from 1-strongly disagree to 7-strongly agree. Higher scores indicated a higher moral disapproval of pornography use (Grubbs, Exline, et al., 2015; Grubbs et al., 2018). The items have shown good psychometric properties in Romanian samples (Huțul & Karner-Huțuleac, 2024a). The internal consistency for moral disapproval of pornography in the present study was excellent (Cronbach’s α = 0.83).

#### Frequency of pornography use

Regarding the frequency of pornography consumption, a single item was used (e.g., “How often have you viewed pornographic materials in the last 12 months?”). Participants rated their responses on an eight-point Likert scale, spanning from 1-never in the past 12 months to 8-once a day or more. This item was provided in other studies regarding the consumption of pornography (Huțul & Karner-Huțuleac, 2023, 2024b). Higher scores indicated a higher frequency of pornography use.

#### Socio-demographic data

The following information was provided by the participants: sex, residence, studies, current marital status, personal income, and religion.

#### Overview of the statistical analysis

First, we assessed the normality of the data distribution using Skewness and Kurtosis coefficients. Then, we examined the association between variables using Pearson correlation. Next, we tested moderated mediation using the SPSS macro PROCESS – Model 7 (Hayes, 2017), with a 95% confidence interval (CI) and 5000 bootstrapped samples to investigate the potential mediation role of difficulties in emotion regulation on the relationship between problematic pornography consumption and somatic symptoms, as well as the possible moderating effect of moral disapproval of pornography on the relationship between problematic pornography consumption and somatic symptoms via difficulties in emotion regulation, while controlling for the frequency of pornography consumption. All statistical analyses were conducted utilizing SPSS software, Version 26 (George & Mallery, 2019).

#### Preliminary data analyses

We computed the Skewness and Kurtosis values to assess the normality of the distribution (Table 2). All the Skewness values were within the 3/-3 limit, indicating normality, as suggested by Hahs-Vaughn and Lomax (2020) and all Kurtosis values values were within 7/-7, indicating normality, as suggested by Hair et al. (2010) and Byrne (2013).

**Table 2.** Descriptive statistics and the association between research variables.

Variables	M	SD	Skewness (SE)	Kurtosis (SE)	1	2	3	4	5
1. Problematic pornography consumption	30.90	18.95	1.97 (.13)	3.57 (.27)	–				
2. Difficulties in emotion regulation	54.92	13.61	.07 (.13)	−.62 (.27)	.20**	–			
3. Somatic symptoms	44.64	17.46	2.27 (.13)	6.64 (.27)	.46**	.27**	–		
4. Frequency of pornography use	3.08	2.09	.75 (.13)	−.51 (.27)	.62**	.05	.28**	–	
5. Moral disapproval of pornography	12.88	7.63	.53 (.13)	−.88 (.27)	.15**	.06	.13*	−.14**	–

*Note:*.

* *p* < .05.

** *p* < .001.

To provide a more detailed picture of somatic symptomatology, descriptive statistics were also examined for somatic symptoms subscales (Table 3). Participants reported higher mean levels of cardio-respiratory response, followed by genitourinary response and gastro-intestinal response, whereas somatic sensitivity and general somatic response were reported at lower levels.

**Table 3.** Descriptive statistics for somatic symptoms subscales.

Variable	Min	Max	Mean	SD
Cardio-respiratory response	11	55	16.26	7.21
Somatic sensitivity	0	13	.38	1.44
Gastro-intestinal response	0	32	1.10	3.15
General somatic response	0	16	.55	1.63
Genitourinary response	3	15	4.42	2.24

#### The association between main variables

The results of the Pearson correlation analysis suggest that problematic pornography consumption is significantly associated with difficulties in emotion regulation, somatic symptoms, frequency of pornography use, and moral disapproval of pornography. Difficulties in emotion regulation correlate significantly and positively with somatic symptoms, but they do not correlate significantly with the frequency of pornography use or with moral disapproval of pornography. Somatic symptoms correlate significantly and positively with both the frequency of pornography use and moral disapproval of pornography. The frequency of pornography use is associated significantly and negatively with moral disapproval of pornography. The results can be observed in Table 2.

#### Testing the indirect effect

**Figure 2.**
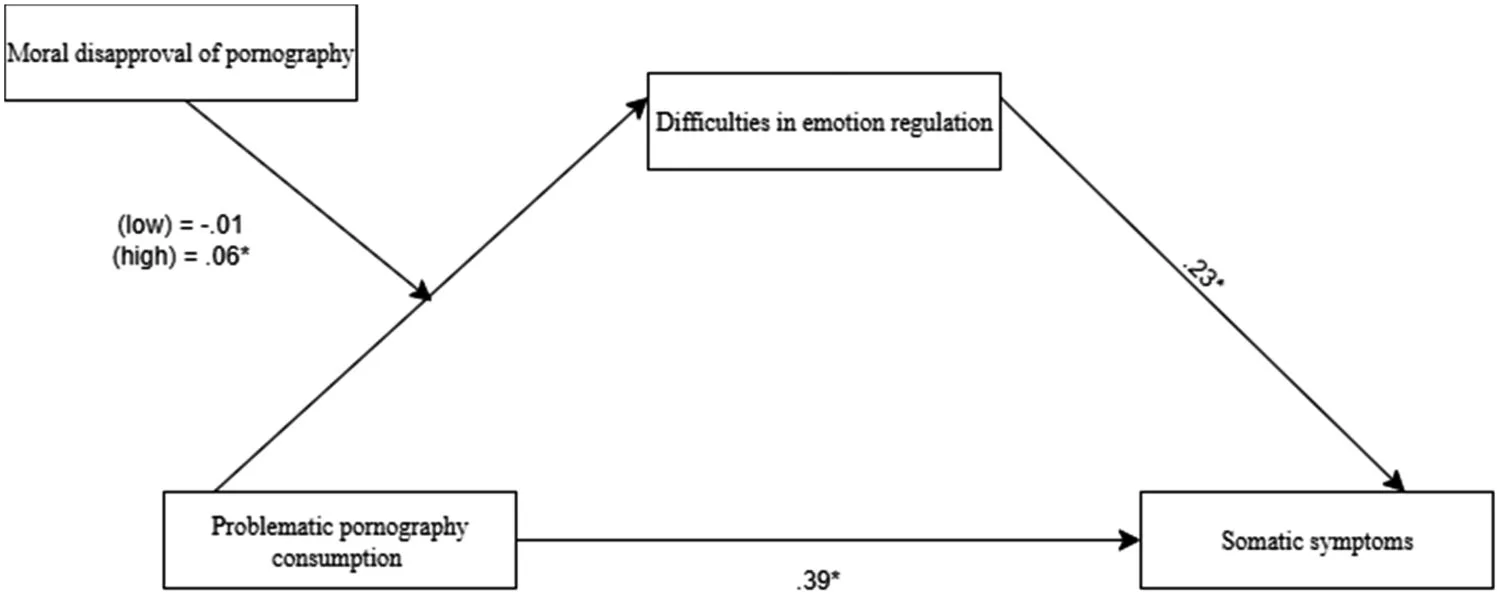
The model of moderated mediation. Note: *p < .001; Values represent unstandardized coefficients.

We used the PROCESS macro in SPSS 26 – Model 7 (Hayes, 2017) to investigate the potential indirect effect of difficulties in emotion regulation on the relationship between problematic pornography consumption and somatic symptoms, as well as the potential moderating effect of moral disapproval of pornography on the relationship between problematic pornography consumption and somatic symptoms via difficulties in emotion regulation, while controlling for the frequency of pornography use (see Figure 2).

The direct effect of problematic pornography consumption on somatic symptoms was significant, *b* = .39; *SE* = .06; *p* < .001; 95% CI [.27; .51]. The conditional indirect effect was significant among participants with high level of moral disapproval of pornography, *b* = .06; *SE* = .02; 95% CI [.03; .11]. The conditional indirect effect was not significant among participants with low level of moral disapproval of pornography, *b*** **=** **−.01; *SE* = .02; 95% CI [−.06; .03], nor among those with average level of moral disapproval of pornography, *b* = .02; *SE* = .01; 95% CI [-.005; .05]. The overall moderated mediation model was supported by the index of moderated mediation = .004, 95% CI [.001; .008].

## Discussion

The present findings reveal several important aspects regarding problematic pornography consumption and the role that somatic symptoms, moral disapproval of pornography, and difficulties in emotion regulation play in this context. The results indicate that problematic pornography consumption is associated with higher levels of somatic symptoms and emotional dysregulation, and that moral disapproval appears to intensify these associations. More specifically, difficulties in emotion regulation emerged as a relevant psychological mechanism linking problematic pornography consumption to somatic symptomatology, particularly among individuals who morally disapprove of pornography use. These findings are in line with previous research suggesting that problematic pornography involvement is often accompanied by emotional dysregulation, psychological distress, and conflicting personal or moral values (Cardoso et al., 2022, 2023; Grubbs et al., 2019).

When faced with distress, some individuals—particularly those who experience difficulties in emotion regulation or engage in problematic patterns of use—may rely on pornography as a maladaptive coping strategy (Borgogna et al., 2022; Bőthe et al., 2021; Huțul et al., 2024; Lewczuk et al., 2020), which, over time, may lead to greater difficulties in emotion regulation and to the reinforcement of avoidance-based mechanisms for dealing with distress and negative emotions (Antons et al., 2023; Brand et al., 2025; Huțul & Karner-Huțuleac, 2023; Starcke et al., 2018). From a coping perspective, somatic symptoms associated with stress or emotional distress may increase vulnerability to engaging in pornography use as a means of short-term relief, potentially reinforcing problematic patterns over time. Emotional dysregulation has also been linked to addictive potential, further strengthening the idea that using pornography as a coping mechanism may lead to problematic and persistent patterns of behavior (Shirazi & Janfaza, 2015; Testa et al., 2024). Although the problematic consumption of pornography has been described as a maladaptive coping mechanism it is also explained by the PPMI model which also links this concept to moral disapproval of pornography as part of moral incongruence (Grubbs et al., 2019).

The main objective of the present study was to address the gap in the literature when it comes to the link between problematic pornography consumption and somatic symptoms. From what we know at the time of the research only particular somatic symptoms were addressed in the literature in connection to PPC, with studies having shown that throbbing headaches, sleep disturbance, cardiopulmonary discomfort were associated with stress and distress and therefore indirectly associated with problematic pornography consumption (Chen et al., 2018; Testa et al., 2024; Zeng et al., 2016; Zheng et al., 2019).

Our study provides significant results regarding the association between problematic pornography consumption and somatic symptoms, that align with the results from few research papers existing on this relationship. Roza et al. (2024) conducted a literature review in which they analyzed 14 studies. Among the main topics were included withdrawal symptoms from pornography and it was stated that around 72.2% of the participants experienced mental, sexual and physical symptoms that were related to pornography consumption. In a similar vein, Mattebo et al., (2018) conducted a longitudinal study among Swedish adolescents and found that higher levels of pornography consumption predicted psychosomatic symptoms such as headaches, stomach-aches, nervousness, irritation and sleep problems. These findings are consistent with the present results and suggest that problematic pornography consumption may be linked to somatic symptoms also among Romanian young adults and adults. Another systemic review states that the association between PPC and somatic symptoms is not “clean-cut” and it’s often mediated by other variables like loneliness (Vieira & Griffiths, 2024) which leads us to believe that there is still a long way to go before we fully understand this topic. Our study addresses an important gap in the literature regarding the direct relationship between PPC and somatic symptoms by examining this relationship in a Romanian sample, and having shown that problematic pornography consumption is associated with higher levels of somatic symptoms. Although there is still much research to be done on this relationship, we believe that this study can provide the starting point for the following studies.

Previous research has found that persistent consumption of pornography is associated with heightened emotional reactivity and lower capacity to manage internal states (Cardoso et al., 2023; Musetti et al., 2022). In other words, individuals who struggle to manage negative emotions have their physiological arousal tend to remain high, which increases the risk of stress-related physical complaints. This interpretation is in line with recent evidence that emotional dysregulation contributes to somatic symptoms by maintaining tension, irritability, and autonomic activation (Qu et al., 2024). Thus, our results suggest that problematic pornography consumption is associated with somatic symptoms both directly and indirectly through difficulties in emotion regulation. Emotion regulation problems appear to intensify the physical manifestations of distress, providing support for the mediating role proposed in our second hypothesis. These findings offer partial support for our second hypothesis, indicating that difficulties in emotion regulation represent a relevant psychological mechanism linking problematic pornography consumption to somatic symptomatology. A central contribution of this study is the finding that at certain levels of moral disapproval, difficulties in emotion regulation indirectly link problematic pornography consumption to somatic symptoms. Moreover, our research has shown that difficulties in emotional regulation had an indirect effect on the relationship between PPC and somatic symptoms but only for the participants who morally disapproved pornography consumption. These findings are fully consistent with our third hypothesis, which proposed that moral disapproval of pornography would strengthen the indirect relationship between problematic pornography consumption and somatic symptoms via difficulties in emotion regulation. The moderated mediation pattern observed in our data confirms that moral disapproval functions as an amplifying factor in this psychological pathway. Taken together, these findings suggest that moral incongruence represents a central component of the proposed model, rather than a secondary or auxiliary variable. Moral disapproval appears to shape not only the emotional correlates of problematic pornography consumption, but also the manner in which psychological distress becomes embodied through somatic symptoms. In this sense, moral incongruence may constitute a key pathway through which internalized moral standards and sociocultural norms translate into both emotional and physical distress. These results align with the literature stating that a higher level of PPC leads to greater difficulties in emotional regulation (Cardoso et al., 2023). It is important to consider that these patterns reinforce the theoretical justification for DERS as a mediator role in the pathway from problematic pornography consumption to somatic symptoms.

This moderating effect is theoretically grounded in the PPMI which suggests that individuals who perceive their pornography use as morally unacceptable experience heightened emotional tension, shame, and internal conflict (Grubbs et al., 2019). These findings should also be interpreted within the Romanian cultural context, where sexuality and pornography tend to be more strongly moralized (Huțul et al., 2025b), potentially intensifying shame-related distress and influencing self-reports of both psychological and somatic symptoms. These reactions appear to intensify both the psychological impact of consumption and its physiological correlates, contributing to the emergence of somatic symptoms. A potential explanation could be represented by the fact that individuals who morally disagree with pornography consumption and are not in line with their values are more likely to experience negative emotions, shame, guilt, and internal conflict, in the moment they engage in it, or after consumption. These moral emotional reactions are intensifying the psychological distress and are reducing the individual’s capacity to regulate their affective states effectively. In contrast, individuals with low moral disapproval are less likely to react with intense negative self-evaluative emotions, in this case, the association is attenuated.

This mechanism is in line with the literature that shows how moral incongruence has a massive role by amplifying the shame and emotional distress in the context of pornography consumption (Grubbs, Exline, et al., 2015; Grubbs et al., 2019; Willoughby & Dover, 2023). Another important result concerns the role of moral disapproval of pornography, which aligns with previous research showing that when individuals consume pornography despite judging it as morally unacceptable, this inconsistency generates shame and psychological distress (Floyd et al., 2020; Lewczuk et al., 2020).

On another note, it is important to acknowledge that the directionality implied by the model proposed in the present study should be interpreted with caution, given the cross-sectional design. Difficulties in emotion regulation may precede and contribute to problematic pornography use rather than result from it, suggesting the possibility of a bidirectional relationship. Such an interpretation is consistent with various studies emphasizing that problematic pornography use is embedded within a broader network of psychological vulnerabilities and diverse coping processes, rather than reflecting a simple linear causal pathway (Bőthe et al., 2024).

In conclusion, our study has shown that difficulties in emotion regulation represent a key mechanism through which problematic pornography consumption contributes to somatic symptoms, particularly among individuals who experience moral incongruence, in accordance with previous literature. These findings extend previous literature by examining these associations in a Romanian sample.

## Limits and future directions

Although the present research offers several important contributions, it is also necessary to acknowledge certain limitations and outline directions for future studies. First, the sample included a considerably higher proportion of women participants (81.0%) compared to men participants (17.4%). This imbalance limits the possibility of drawing meaningful gender-based comparisons and may restrict the generalizability of the findings. Due to the disproportionate sex distribution, it was not statistically appropriate to explore or test gender differences in problematic pornography consumption or in the proposed model, and therefore such analyses were not conducted. As a result, the present findings should be interpreted with caution, particularly when considering their applicability to male populations, who may experience higher levels or different patterns of PPC-related concerns. Future research would benefit from recruiting more balanced samples or from intentionally examining sex differences in problematic pornography consumption, emotion regulation, and somatic responses.

Second, all variables were measured through self-report instruments. While widely used in psychological research, self-report measures are vulnerable to social desirability biases, recall biases, and subjective interpretation. This concern is especially relevant in studies addressing morally sensitive topics such as pornography consumption, where participants might underreport or misrepresent their behavior. Future studies could incorporate more objective measures, such as behavioral data, clinician-rated assessments, or physiological indicators of somatic responses, in order to provide a more comprehensive perspective.

Third, the cross-sectional design does not allow for causal inferences regarding the direction of the relationships among problematic pornography consumption, emotion regulation difficulties, and somatic symptoms. Longitudinal or experimental designs would enable a more robust examination of temporal sequencing and causality, offering deeper insight into whether problematic pornography consumption contributes to emotional dysregulation and somatic manifestations over time, or whether preexisting emotional vulnerabilities increase susceptibility to problematic use. Moreover, future studies may consider including additional psychological variables—such as anxiety, depression, or stress sensitivity—which are known to influence both emotion regulation processes and somatic symptom expression. Incorporating these factors may help clarify the unique contribution of problematic pornography consumption within a more complex psychosocial context.

Finally, it is important to consider that the present research relied on a sample drawn exclusively from Romania, within which a substantial proportion of participants identified their religion as Eastern Orthodox Christianity (60.7%). Given the central role that moral and religious norms play in the formation and development of attitudes toward pornography, the associations identified in the present work—particularly those involving moral disapproval—should be interpreted with the acknowledgment that they may partially reflect processes specific to the cultural and religious context in which the study was conducted. Accordingly, the generalizability of the findings to other cultural contexts or to populations with different religious compositions may be limited. Consequently, future research could seek to replicate the proposed model across a broader range of cultural and religious settings, thereby allowing for the consideration of factors such as religiosity or religious affiliation as additional variables.

Despite these limitations, the present findings provide an important foundation for future research, highlighting the relevance of somatic symptoms in the context of problematic pornography consumption and underscoring the psychological mechanisms through which this association unfolds.

## Theoretical and practical implications

From a theoretical perspective, the present study offers insights that may extend current knowledge within the framework of the PPMI. While the PPMI primarily focuses on moral and emotional processes, our findings suggest that somatic responses may also be relevant in understanding the broader experience of problematic pornography consumption. By incorporating somatic symptoms into this conceptual landscape, the study highlights the possibility that problematic pornography consumption may be embedded in a more complex biopsychosocial process than previously emphasized. Additionally, the results indicate that emotional dysregulation may function as a psychological mechanism linking pornography-related distress to somatic manifestations. This observation aligns with existing theories emphasizing the role of emotion regulation difficulties in stress responses and suggests that future theoretical models might benefit from more explicit consideration of physiological or somatic outcomes associated with problematic pornography behavior.

The moderated mediation pattern observed in this study further suggests that moral disapproval may amplify the indirect association between problematic pornography consumption and somatic symptoms through emotional dysregulation. In this regard, the findings provide preliminary support for the idea that moral incongruence may intensify both emotional and physiological discomfort, complementing and expanding previous conceptualizations of the PPMI model. Overall, the present study offers a more integrative view of problematic pornography consumption by bringing together moral, emotional, and somatic components, and may serve as a foundation for future theoretical refinements.

From a practical standpoint, the findings may hold relevance for mental health professionals working with individuals experiencing difficulties related to pornography consumption. The association identified between problematic pornography consumption, emotional dysregulation, and somatic symptoms suggests that clinicians may benefit from assessing somatic complaints as part of a broader emotional and behavioral profile. Somatic symptoms may serve as indirect indicators of emotional distress or unresolved internal conflicts rather than purely medical issues. Given the potential mediating role of emotion regulation difficulties, interventions that focus on enhancing emotional awareness, distress tolerance, and adaptive regulation strategies—such as those found in dialectical behavior therapy, mindfulness-based approaches, or affect-regulation interventions—may be particularly useful for individuals who struggle with problematic pornography consumption.

Furthermore, the moderating role of moral disapproval underscores the importance of adopting culturally and morally sensitive approaches in clinical practice. These findings position moral incongruence as a clinically and socially relevant mechanism, highlighting that pornography-related distress may emerge not only from individual behavior patterns, but also from internalized moral norms and broader sociocultural narratives surrounding sexuality. In contexts such as Romania, where pornography use is often accompanied by stronger moral or religious norms, individuals may experience heightened shame, guilt, or internal conflict. Such emotional reactions may exacerbate somatic symptoms and hinder therapeutic progress if left unaddressed. Clinicians may therefore consider integrating discussions about personal values, moral beliefs, and perceived incongruence into treatment, using approaches that reduce shame and encourage self-compassion. At the same time, it is important to acknowledge that moral incongruence and the distress associated with it are not solely individual phenomena, but are often shaped by broader social, cultural, and normative discourses surrounding sexuality (Grubbs et al., 2019; Grubbs & Perry, 2019; Huțul et al., 2025b; 2026; Vaillancourt-Morel & Bergeron, 2019). Accordingly, preventive and psychoeducational efforts may also benefit from addressing these issues at a societal level, for example through public health initiatives, educational programs, or efforts aimed at reducing stigma related to sexuality and pornography use.

Finally, these findings may be useful for psychoeducation and prevention efforts, by raising awareness about the potential interplay between emotional distress, moral conflict, and somatic complaints in the context of problematic pornography consumption.

## Data Availability

The data supporting the findings of this research are available from the corresponding author upon request.
